# Salivary IgAs and Their Role in Mucosal Neutralization of SARS-CoV-2 Variants of Concern

**DOI:** 10.1128/jcm.01065-22

**Published:** 2022-08-29

**Authors:** Gabriel Diem, Eliott Lafon, Angelika Bauer, Cornelia Lass-Flörl, Markus Reindl, Doris Wilflingseder, Wilfried Posch

**Affiliations:** a Institute of Hygiene and Medical Microbiology, Medical University of Innsbruck, Innsbruck, Austria; b Clinical Department of Neurology, Medical University of Innsbruck, Innsbruck, Austria; Cepheid

**Keywords:** SARS-CoV-2, mucosal immunity, neutralizing antibodies, salivary IgA, vaccines, variants of concern, virus neutralization

## LETTER

Rapid emergence of novel SARS-CoV-2 variants and rising incidences despite vaccination demands detailed understanding of vaccine- or vaccine/disease-induced protection (1, 2). Most studies investigated immunity in sera; however, little is known about neutralizing IgAs in the mucosa after intramuscular COVID-19 vaccination, which are associated with protection (3, 4). We recently discovered that mRNA-based vaccines could induce significantly higher levels of serum IgAs (5). Here, we investigated serum IgG, IgA and salivary IgA titers, as well as serum and saliva neutralization of fully vaccinated and heterologous (2xChAdOx1/mRNA, *n* = 31; see Table S1 in the supplemental material) or homologous boostered (3xmRNA, *n* = 20; Table S2) or fully vaccinated and non-Omicron convalescent (2xVac/Conv, *n* = 20; Table S3) subjects. Sera were analyzed for SARS-CoV-2 receptor binding domain (RBD)-specific IgGs, using a quantitative Conformite Europeenne *In-Vitro* Diagnostic (CE-IVD)-certified chemiluminescent microparticle immunoassay (CMIA), or for SARS-CoV-2 S1-specific IgAs, using a semiquantitative CE-IVD-certified enzyme-linked immunosorbent assay (ELISA). In addition, S1-specific IgA titers were determined in saliva samples. Neutralization assays using sera and saliva were performed with replication-competent SARS-Cov-2 variants. We found a broad range of serum and salivary IgA titers in the cohorts tested and highlighted heterogeneous mucosal neutralization against variants of concern (VOCs) depending on previous vaccination and infection.

**FIG 1:**
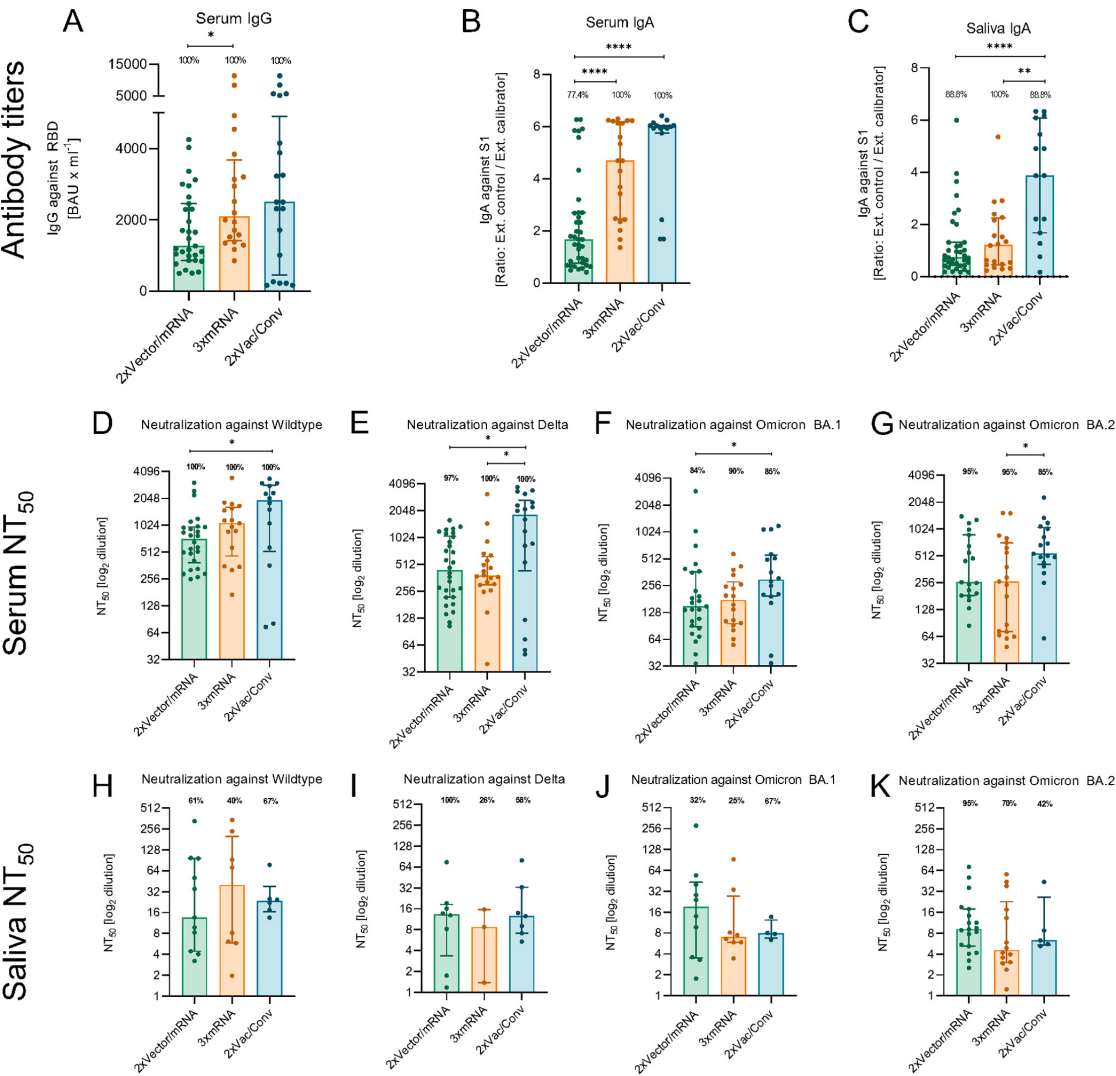
Analyses of SARS-CoV-2 antibody titers and neutralization capacity using sera and saliva from vaccinated and convalescent patients. Analysis of antibody titers of twice vector and once mRNA boostered (2xVector/mRNA, green), triple mRNA vaccinated (3xmRNA, orange), and twice vaccinated and non-Omicron convalescent patients (2xVac/Conv, blue) are shown. (A to C) Serum (A) was tested for SARS-CoV-2 IgG-specific IgG titers, and serum (B) and saliva (C) were tested for the presence of SARS-CoV-2 S1-specific IgA. Percentages above bars indicates the fraction of positive titers (>7.1 binding antibody units [BAU] · mL^−1^ for IgG and ratio >1.1 for IgA). (D to G) Half maximum neutralization (NT_50_) titers are shown for serum against SARS-CoV-2 wild-type, Delta, and Omicron BA.1 and BA.2 variants. (H to K) NT_50_ titers of saliva are presented against SARS-CoV-2 wild-type, Delta, and Omicron BA.1 and BA.2 variants. Percentages above bars indicate the fraction of positive neutralization titers (NT_50_ > 32 for serum, NT_50_ > 1 for saliva). Statistical significance between the groups was determined by Mann-Whitney U test for nonparametric distribution (*, *P* < 0.05; **, *P* < 0.01; ***, *P* < 0.001; ****, *P* < 0.0001). Medians are visualized as bars together with the interquartile range as error bars.

All cohorts tested positive for serum IgGs as well as serum and salivary IgAs, with the highest titers detected in convalescent individuals (Fig. 1A to C, Table 1). Next, we investigated serum half-maximum neutralization titers against replication-competent SARS-CoV-2 wild-type virus (WT) as well as Delta and Omicron BA.1 (BA.1) and BA.2 (BA.2) variants (Fig. 1D to G). The convalescent cohort showed strongest serum neutralization against all tested viruses compared to heterologous and homologous vaccinees (Fig. 1D to G, Table 2, in serum). Overall, serum neutralization revealed that both Omicron subvariants could escape more effectively; however, BA.1 was superior compared to BA.2 (Fig. 1F and G). Effective salivary neutralization against WT, Delta, BA.1, and BA.2 ranged between 40 and 67%, 26 and 100%, 25 and 67%, and 42 and 95%, respectively (Fig. 1H to K). No significant differences in salivary neutralization of the tested variants were found between the cohorts (Fig. 1H to K). The heterologous cohort illustrated neutralizing activity in the saliva mainly against Delta and BA.2, while convalescent individuals exceeded against WT and BA.1 (Fig. 1H to K). In contrast, the homologous cohort showed the lowest percentages of effective neutralizers against WT, Delta, and BA.1 and reached the lowest neutralizing abilities against VOCs (Fig. 1I to K, Table 2, in saliva). Among all, heterologous vaccinees were superior in neutralization against Delta and both Omicron subvariants, while convalescent individuals exhibited higher protection against Delta and BA.2 (Fig. 1H to K). Essentially, we demonstrated for the first time that dependent on the COVID-19 vaccines, a broad range of salivary IgAs and a heterogeneous mucosal neutralization against current VOCs were detected. These findings may partially explain the current epidemiological Omicron situation with increasing numbers of break-through infections among vaccinated individuals, but the exact cutoff values for serum and salivary neutralizing antibodies need to be determined in future clinical studies (6, 7). In addition, serum IgGs correlate with serum neutralization, but no such conclusions can be drawn for salivary IgAs and mucosal immunity after intramuscular vaccination (Fig. S1). Potential limitations of our study might be the combined evaluation of BNT162b2 and mRNA-1273 vaccine recipients as well as the sampling day after last immunization. However, our findings on salivary immunity support the idea of an adapted vaccine or alternative delivery strategy such as mucosal vaccine administration as a booster for optimal protection against SARS-CoV-2 (3, 8).

**TABLE 1:** Median antibody titers of Serum IgG against SARS-CoV-2 RBD in BAU·mL^−1^ and median antibody titers for SARS-CoV-2 S1 IgA in serum and saliva are illustrated as ratio (external control/external calibrator)

Ig isotype and target region	Group	Median IgG or IgA titer (CI 95%)^*a*^
Serum IgG (against RBD)	2xVector/mRNA	1,373 (1,041–2,072)
	3xmRNA	2,103 (1,527–3,206)
	2xVac/Conv	2,501 (1,017–4,156)
Serum IgA (against S1)	2xVector/mRNA	1.770 (1.070–2.330)
	3xmRNA	4.690 (2.470–6.130)
	2xVac/Conv	5.990 (5.750–6.040)
Saliva IgA (against S1)	2xVector/mRNA	0.7300 (0.5400–1.010)
	3xmRNA	1.220 (0.4700–2.160)
	2xVac/Conv	3.870 (1.680–6.080)

a IgG titers, BAU/mL; IgA titers, ratio (ext. control/ext. calibrator); CI, confidence interval.

**TABLE 2:** Median NT_50_ values of 2xVector/mRNA, 3xmRNA and 2xVac/Conv groups with 95% confidence interval (CI 95%)

Virus variant	Group	Median (CI 95%)
In serum		
NT_50_ against wild type	2xVector/mRNA	719.1 (493–962.8)
	3xmRNA	1,076 (571.4–1,579)
	2xVac/Conv	1,931 (360.9–3,035)
NT_50_ against Delta	2xVector/mRNA	439.4 (263.9–821.6)
	3xmRNA	381.2 (306.8–564)
	2xVac/Conv	1,834 (539.9–2,524)
NT_50_ against Omicron (BA.1)	2xVector/mRNA	149.4 (184.1–879.3)
	3xmRNA	175.8 (97.03–264.4)
	2xVac/Conv	297.3 (193.2–575.7)
NT_50_ against Omicron (BA.2)	2xVector/mRNA	259.7 (184.1–879.3)
	3xmRNA	263.5 (72.39–717.5
	2xVac/Conv	539.7 (429.6–1,028)
In saliva		
NT_50_ against wild type	2xVector/mRNA	13.58 (4.01–97.91)
	3xmRNA	39.65 (1.96–345.2)
	2xVac/Conv	23.25 (13.49–77.79)
NT_50_ against Delta	2xVector/mRNA	13.29 (1.17–75.05)
	3xmRNA	8.71 (1.38–15.64)
	2xVac/Conv	12.56 (5.38–79.21)
NT_50_ against Omicron (BA.1)	2xVector/mRNA	19.03 (–14.6–106.9)
	3xmRNA	7.01 (3.43–92.32)
	2xVac/Conv	7.89 (6.30–13.69)
NT_50_ against Omicron (BA.2)	2xVector/mRNA	9.1 (5.18–17.79)
	3xmRNA	4.52 (2.94–38.32)
	2xVac/Conv	6.29 (5.21–43.77)
