# Supplementary material for: Salivary IgAs and Their Role in Mucosal Neutralization of SARS-CoV-2 Variants of Concern
Source: J Clin Microbiol. 2022 Aug 29;60(9):e01065-22. doi: 10.1128/jcm.01065-22 (PMC9491179; doi:10.1128/jcm.01065-22)
Supplement: Supplemental file 1 — Supplemental material. Download jcm.01065-22-s0001.pdf, PDF file, 1.0 MB [file jcm.01065-22-s0001.pdf]

Figure S1

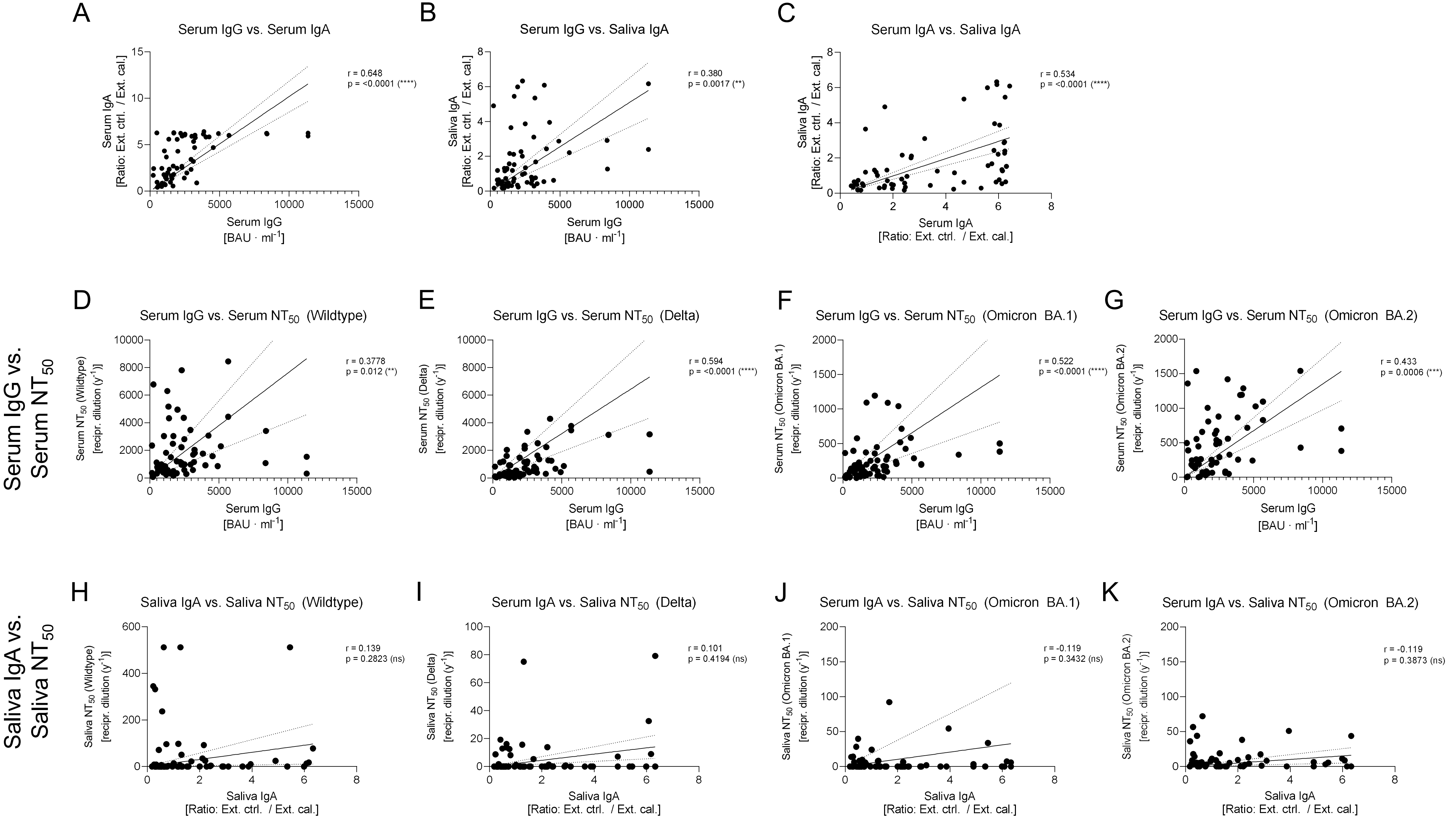
A
Serum IgG vs. Serum IgA
Serum IgA
[Ratio: Ext. ctrl. / Ext. cal.]
Serum IgG
[BAU · ml^{-1}]
r = 0.648
p = <0.0001 (****)
B
Serum IgG vs. Saliva IgA
Saliva IgA
[Ratio: Ext. ctrl. / Ext. cal.]
Serum IgG
[BAU · ml^{-1}]
r = 0.380
p = 0.0017 (**)
C
Serum IgA vs. Saliva IgA
Saliva IgA
[Ratio: Ext. ctrl. / Ext. cal.]
Serum IgA
[Ratio: Ext. ctrl. / Ext. cal.]
r = 0.534
p = <0.0001 (****)
Serum IgG vs. Serum NT_{50}
D
Serum IgG vs. Serum NT_{50} (Wildtype)
Serum NT_{50} (Wildtype)
[recipr. dilution (y^{-1})]
Serum IgG
[BAU · ml^{-1}]
r = 0.3778
p = 0.012 (**)
E
Serum IgG vs. Serum NT_{50} (Delta)
Serum NT_{50} (Delta)
[recipr. dilution (y^{-1})]
Serum IgG
[BAU · ml^{-1}]
r = 0.594
p = <0.0001 (****)
F
Serum IgG vs. Serum NT_{50} (Omicron BA.1)
Serum NT_{50} (Omicron BA.1)
[recipr. dilution (y^{-1})]
Serum IgG
[BAU · ml^{-1}]
r = 0.522
p = <0.0001 (****)
G
Serum IgG vs. Serum NT_{50} (Omicron BA.2)
Serum NT_{50} (Omicron BA.2)
[recipr. dilution (y^{-1})]
Serum IgG
[BAU · ml^{-1}]
r = 0.433
p = 0.0006 (***)
Saliva IgA vs. Saliva NT_{50}
H
Saliva IgA vs. Saliva NT_{50} (Wildtype)
Saliva NT_{50} (Wildtype)
[recipr. dilution (y^{-1})]
Saliva IgA
[Ratio: Ext. ctrl. / Ext. cal.]
r = 0.139
p = 0.2823 (ns)
I
Serum IgA vs. Saliva NT_{50} (Delta)
Saliva NT_{50} (Delta)
[recipr. dilution (y^{-1})]
Saliva IgA
[Ratio: Ext. ctrl. / Ext. cal.]
r = 0.101
p = 0.4194 (ns)
J
Serum IgA vs. Saliva NT_{50} (Omicron BA.1)
Saliva NT_{50} (Omicron BA.1)
[recipr. dilution (y^{-1})]
Saliva IgA
[Ratio: Ext. ctrl. / Ext. cal.]
r = -0.119
p = 0.3432 (ns)
K
Serum IgA vs. Saliva NT_{50} (Omicron BA.2)
Saliva NT_{50} (Omicron BA.2)
[recipr. dilution (y^{-1})]
Saliva IgA
[Ratio: Ext. ctrl. / Ext. cal.]
r = -0.119
p = 0.3873 (ns)

Table S1: Individuals immunized 2x with vector and 1x with mRNA vaccine

ID	Sex	Age	1. Vaccination	2. Vaccination	3. Vaccination	Days after last immunization	COVID19
V1	F	26	ChAdOx1	ChAdOx1	BNT162b2	44	no
V2	F	52	ChAdOx1	ChAdOx1	BNT162b2	47	no
V3	F	58	ChAdOx1	ChAdOx1	BNT162b2	52	no
V4	F	29	ChAdOx1	ChAdOx1	BNT162b2	26	no
V5	M	48	ChAdOx1	ChAdOx1	BNT162b2	55	no
V6	F	48	ChAdOx1	ChAdOx1	BNT162b2	56	no
V7	F	29	ChAdOx1	ChAdOx1	BNT162b2	47	no
V8	M	28	ChAdOx1	ChAdOx1	BNT162b2	48	no
V9	F	59	ChAdOx1	ChAdOx1	BNT162b2	54	no
V10	M	36	ChAdOx1	ChAdOx1	BNT162b2	28	no
V11	M	43	ChAdOx1	ChAdOx1	BNT162b2	56	no
V12	F	54	ChAdOx1	ChAdOx1	BNT162b2	55	no
V13	M	57	ChAdOx1	ChAdOx1	BNT162b2	60	no
V14	M	62	ChAdOx1	ChAdOx1	mRNA-1273	62	no
V15	F	58	ChAdOx1	ChAdOx1	BNT162b2	170	no
V16	F	52	ChAdOx1	ChAdOx1	BNT162b2	56	no
V17	M	30	ChAdOx1	ChAdOx1	BNT162b2	56	no
V18	F	57	ChAdOx1	ChAdOx1	mRNA-1273	43	no
V19	M	29	ChAdOx1	ChAdOx1	BNT162b2	49	no
V20	F	51	ChAdOx1	ChAdOx1	mRNA-1273	64	no
V21	M	42	ChAdOx1	ChAdOx1	BNT162b2	63	no
V22	F	57	ChAdOx1	ChAdOx1	mRNA-1273	55	no
V23	M	62	ChAdOx1	ChAdOx1	mRNA-1273	43	no
V24	F	51	ChAdOx1	ChAdOx1	BNT162b2	43	no
V25	F	39	ChAdOx1	ChAdOx1	BNT162b2	25	no
V26	F	35	ChAdOx1	ChAdOx1	BNT162b2	60	no
V27	F	32	ChAdOx1	ChAdOx1	mRNA-1273	56	no
V28	F	46	ChAdOx1	ChAdOx1	BNT162b2	44	no
V29	F	27	ChAdOx1	ChAdOx1	BNT162b2	57	no
V30	F	57	ChAdOx1	ChAdOx1	BNT162b2	57	no
V31	F	51	ChAdOx1	ChAdOx1	BNT162b2	57	no

n = 31
Age, median (IQR): 48 (25)
Sex ratio (M/F): 32% / 68%
Days after last immunization (geometric mean): 51.35

Table S2: Individuals immunized 3x with mRNA vaccine

ID	Sex	Age	1. Vaccination	2. Vaccination	3. Vaccination	Days after last immunization	COVID19
M1	F	37	BNT162b2	BNT162b2	BNT162b2	57	no
M2	F	27	BNT162b2	BNT162b2	mRNA-1273	15	no
M3	F	54	BNT162b2	BNT162b2	BNT162b2	53	no
M4	F	35	mRNA-1273	mRNA-1273	BNT162b2	47	no
M5	M	32	mRNA-1273	mRNA-1273	mRNA-1273	58	no
M6	M	32	mRNA-1273	mRNA-1273	mRNA-1273	58	no
M7	M	29	BNT162b2	BNT162b2	BNT162b2	88	no
M8	M	29	BNT162b2	BNT162b2	BNT162b2	88	no
M9	F	44	BNT162b2	BNT162b2	BNT162b2	48	no
M10	F	44	BNT162b2	BNT162b2	BNT162b2	48	no
M11	M	26	BNT162b2	BNT162b2	BNT162b2	55	no
M12	F	28	BNT162b2	BNT162b2	BNT162b2	50	no
M13	F	23	BNT162b2	BNT162b2	BNT162b2	36	no
M14	F	25	BNT162b2	BNT162b2	BNT162b2	35	no
M15	F	26	BNT162b2	BNT162b2	BNT162b2	58	no
M16	F	56	mRNA-1273	mRNA-1273	mRNA-1273	8	no
M17	M	52	mRNA-1273	mRNA-1273	BNT162b2	43	no
M18	F	50	mRNA-1273	mRNA-1273	mRNA-1273	16	no
M19	F	17	BNT162b2	BNT162b2	BNT162b2	14	no
M20	M	17	BNT162b2	BNT162b2	BNT162b2	14	no

n = 20
Age, median (IQR): 31.5 (16.45)
Sex ratio (M/F): 35% / 65%
Days after last immunization (geometric mean): 35.91

Table S3: Individuals immunized 2x and convalescent (non-Omicron)

ID	Sex	Age	1. Vaccination	2. Vaccination	Days between 2nd vaccination and infection	Days after infection	COVID19
C1	F	36	ChAdOx1	ChAdOx1	258	58	Delta
C2	M	54	BNT162b2	BNT162b2	156	160	WT
C3	F	21	ChAdOx1	ChAdOx1	68	190	Alpha
C4	F	26	BNT162b2	BNT162b2	210	35	Delta
C5	M	27	ChAdOx1	ChAdOx1	210	35	Delta
C6	F	28	ChAdOx1	BNT162b2	175	15	WT
C7	F	28	ChAdOx1	ChAdOx1	132	177	Alpha
C8	F	34	ChAdOx1	ChAdOx1	198	25	Delta
C9	M	24	BNT162b2	BNT162b2	194	22	Delta
C10	M	33	BNT162b2	BNT162b2	262	54	Delta
C11	M	20	BNT162b2	BNT162b2	163	14	Delta
C12	M	67	BNT162b2	BNT162b2	186	38	Delta
C13	F	21	ChAdOx1	ChAdOx1	68	190	Alpha
C14	F	31	ChAdOx1	ChAdOx1	79	70	Delta
C15	F	33	BNT162b2	BNT162b2	239	19	Delta
C16	M	27	ChAdOx1	ChAdOx1	223	35	Delta
C17	F	28	ChAdOx1	ChAdOx1	167	15	WT
C18	F	28	ChAdOx1	BNT162b2	139	177	Alpha
C19	F	34	ChAdOx1	ChAdOx1	233	25	Delta
C20	M	30	ChAdOx1	ChAdOx1	295	21	Delta

n = 20
Age, median (IQR): 28 (7.5)
Sex ratio (M/F): 40% / 60%
Days between 2nd vaccination and infection (geometric mean): 169.62
Days after infection (geometric mean): 44.92

ABBREVIATIONS

Ab	Antibody
BA.1	SARS-CoV-2 Omicron variant subtype BA.1
BA.2	SARS-CoV-2 Omicron variant subtype BA.2
BAU	Binding antibody unit
BNT162b2	BioNTech/Pfizer COVID-19 vaccine
ChAdOx1	AstraZeneca COVID-19 vaccine
CI	Confidence interval
COVID-19	Coronavirus disease 2019
Ig	Immunglobulin
IgA	Immunglobulin A
IgG	Immunglobulin G
IQR	Interquartile range
mRNA	Messenger ribonucleic acid
mRNA-1273	Moderna COVID-19 vaccine
NT_{50}	Half-maximum neutralization titer
RBD	Receptor binding domain
S1	SARS-CoV-2 spike protein region 1
SARS-CoV-2	Severe acute respiratory syndrome coronavirus type 2
VOC	Variants of concern
WT	Wildtype
2xVector/mRNA	group of individuals vaccinated with 2x ChAdOx1 and boostered with mRNA-based SARS-CoV-2 vaccine
3xmRNA	group of individuals vaccinated with 3x mRNA-based SARS-CoV-2 vaccine
2xVac/Conv	group of COVID-19 convalescent vaccinated with 2x SARS-CoV-2 vaccine (vector or RNA-based)

METHODS

Ethics Statement

Written informed consent was obtained from all donors of leftover nasopharyngeal/ oropharyngeal specimens, serum and saliva samples by the participating clinics. The Ethics Committee of the Medical University of Innsbruck approved the use of anonymized leftover specimens of COVID-19 patients (ECS1166/2020) and healthy donors (ECS1166/2018) for scientific purposes.

Human samples

In this study, serum and saliva samples of 71 individuals vaccinated twice with vector-based vaccine ChAdOx1 and once mRNA (BNT162b2 or mRNA-1273) vaccine boostered (2xVec/mRNA), n=31 (Table S1), triple mRNA vaccinated individuals (3xmRNA; homologous and heterologous with either BNT162b2 or mRNA-1273), n=20 (Table S2) and twice vaccinated (ChAdOx1, BNT162b2 or mRNA-1273) and non-Omicron convalescent patients

(2xVac/Conv), n=20 (Table S3) were collected. The median age of participants was 48 years (26 – 62 years) for the 2xVec/mRNA group, 31.5 years (17 – 56 years) for the 3xmRNA group and 28 years (20 – 67 years) for the 2xVac/Conv group. The percentage of male and female patients of 2xVec/mRNA, 3xmRNA and 2xVac/Conv included in the study was 32%/68%, 35%/65% and 40%/60% respectively. The median sampling days after last immunization of these 3 cohorts were day 55 (2xVector/mRNA), day 47.5 (3xmRNA) and day 56 (2xVac/Conv) (Tables S1-3). All included COVID-19 patients were diagnosed by PCR and showed mild disease severity, which did not require any treatment or hospitalization (Table S3). Serum samples from vaccinated or COVID-19 convalescent participants were retrieved from blood samples in serum collection tubes by centrifugation at 300 g for 5 min and serum fractions were carefully collected. Saliva samples were collected using Saliva collection tubes Salivette (Sarstedt, Nümbrecht, Germany). As suggested by the manufacturer, the liquid phase was obtained after centrifugation at 4000 x g for 5 minutes and stored at -80°C until use. Both, serum and saliva samples were incubated for 1h at 56 °C to inactivate complement components that could interfere with subsequent assays. All samples were stored at -80°C until use.

Viruses

Clinical specimens for SARS-CoV-2 Delta (B.1.617.2) Omicron (B.1.1.529) BA.1 and BA.2 were isolated from COVID-19 positive swabs (Ethics statement, ECS1166/2020) and cultured as previously described(1). SARS-CoV-2 Wildtype virus (WT) was obtained from a repository (BEI Resources, Manassas, VA, USA; CFAR/NIBSC; Nr-52281) and propagated according to the manufacturer's instructions.

CMIA/ELISA

Sera were analyzed with the quantitative CE-IVD certified SARS-CoV-2 IgG II Quant Assay (Abbott, USA). The chemiluminescent microparticle immunoassay (CMIA) SARS-CoV-2-IgG-II-Quant-Assay was performed in order to assess anti-SARS-CoV-2 IgG against RBD. CMIA results were calculated to $BAU \cdot ml^{-1}$ according to manufacturer instructions and the cut-off value for positive results was set at 7.1 $BAU \cdot ml^{-1}$. IgA antibody titers in Sera and Saliva were analyzed using the semi-quantitative CE-IVD certified Anti-SARS-CoV-2 IgA against S1 ELISA (Euroimmun, Lübeck, Germany). Results are shown as ratio (external control/external calibrator) the manufacturer defines a ratio ≥ 1.1 as positive.

Immunofluorescence neutralization assay

VeroE6/TMPRSS2 cells ($2 \cdot 10^4$) were seeded in a 96-well plate with culture medium (DMEM high glucose medium supplemented with 10% FCS, 1% L-Glutamine, 1%

Penicillin/Streptomycin; all reagents were obtained from Sigma Aldrich, St. Louis, MO, USA) and incubated overnight at 37 °C and 5% CO_2. After 2 days in culture, heat-inactivated serum and saliva samples were serial-diluted from 1:8 to 1:4096 or 1:4 to 1:512, respectively. Dilutions were incubated with SARS-CoV-2 Wildtype or variant strains ($2.5 \cdot 10^2$ $PFU \cdot ml^{-1}$) for 1 h at 37 °C and subsequently dilutions were inoculated with VeroE6/TMPRSS2 1h at 37 °C and 5% CO_2. After incubation, inoculum was aspirated, cells washed with D-PBS and incubated in DMEM supplemented with 1.5% FCS, 1% L-Glutamine and 1% Penicillin/Streptomycin at 37°C and 5% CO_2. After 16 h, medium was removed and cells fixed in 4% Formaline solution (Sigma Aldrich, St. Louis, MO, USA) for 30 minutes at room temperature. After fixation, cells were permeabilized (Intracellular Staining Permeabilization Wash Buffer, Biolegend, California), according to the manufacturer's instructions. The same buffer was used for immunofluorescence staining with primary (monoclonal rabbit- anti SARS-CoV-2 nucleocapsid antibody, Sinobiological, China) and secondary (Goat anti-Rabbit IgG Alexa Fluor 647, Invitrogen, USA) antibodies. Cells were washed twice in D-PBS before imaging on the Operetta CLS^{TM} Microscope (Perkin Elmer, Waltham, MA, USA). Image analysis and quantification of infected areas was performed using Harmony software 4.8 (Perkin Elmer, Waltham, MA, USA). Half-maximum neutralizing capacity (NT_{50}) values from neutralization curves were calculated using four-parameter nonlinear regression in GraphPad Prism v.9. As cut-off values for positive neutralization 1:32 for serum and 1:1 for saliva was defined.

Statistical analysis

Statistical analysis was performed using GraphPad Prism v.9. Statistical significance of SARS-CoV-2-specific antibody ratio and NT_{50} levels were determined using Mann-Whitney-U test for nonparametric distribution (GraphPad Prism).

Acknowledgements

We thank our technicians Sophie Ann Erckert, Ruth Mader; Bettina Sartori (Institute of Hygiene and Medical Microbiology, Medical University of Innsbruck, Innsbruck, Austria) and Dagmar Rudzki (Clinical Department of Neurology, Medical University of Innsbruck, Innsbruck, Austria) as well as Michael Jäger, MSc (Institute of Hygiene and Medical Microbiology, Medical University of Innsbruck, Innsbruck, Austria) and Prof. Dr. Rosa Bellmann-Weiler (Department of Internal Medicine II, Medical University of Innsbruck, Innsbruck, Austria) for their valuable help and support regarding this study. SARS-CoV-2 RNA (NIBSC 19/304) was obtained from the National Institute for Biological Standards and Control, UK. : SARS-Related Coronavirus 2, Isolate USA-WA1/2020 NR-5228 was deposited by the Centers for Disease Control and

Prevention and obtained through BEI Resources, NIAID, NIH. The authors were supported by the Austrian Science Fund (FWF; P34070-B13 to W.P. and P33510-B13 to D.W.), the Anniversary Fund of the Austrian National Bank (OeNB; P17614 to W.P., P17633 to D.W.) and the State of Tyrol (No. 70454 to W.P.).

Figure S1: Correlations of antibody titers and neutralization capacity of serum and saliva.

The graphs show correlations and simple linear regressions of serum IgG against serum IgA **(A**) and saliva IgA **(B)**, as well as serum IgA against saliva IgA **(C)**. Correlations and simple linear regression of Serum IgG against serum NT_{50} values for SARS-CoV-2 Wildtype, Delta, Omicron BA.1 and BA.2 were analyzed **(D-G)**. Furthermore, correlations were performed of saliva IgA against saliva NT_{50} values for SARS-CoV-2 Wildtype, Delta, Omicron BA.1 and BA.2 **(H-K)**. Significances between the groups were determined with a non-parametric spearman correlation using GraphPad Prism v.9 (GraphPad Prism; ns: p0.1234; *: p<0.0332; **: p<0.0021; ***: p<0.0002; ****: p<0.0001).

TABLE LEGENDS

Table S1: Characteristics from 2x Vector and 1x mRNA vaccinated individuals (n=31)

Sex, age, days after last immunization and convalescence status are presented.

Table S2: Characteristics from 3x mRNA vaccinated individuals (n=20)

Sex, age, days after last immunization and convalescence status are shown.

Table S3: Characteristics from 2x vaccinated and convalescent individuals (n=20)

Sex, age, days after last immunization (including diseased) and convalescence status with the respective virus variant are demonstrated. (vac. = vaccinated, conv = convalescent).

REFERENCES

1. Lafon E, Jager M, Bauer A, Reindl M, Bellmann-Weiler R, Wilflingseder D, Lass-Florl C, Posch W. 2022. Comparative analyses of IgG/IgA neutralizing effects induced by three COVID-19 vaccines against variants of concern. J Allergy Clin Immunol doi:10.1016/j.jaci.2022.01.013.
